# Physical Literacy Knowledge Questionnaire: feasibility, validity, and reliability for Canadian children aged 8 to 12 years

**DOI:** 10.1186/s12889-018-5890-y

**Published:** 2018-10-02

**Authors:** Patricia E. Longmuir, Sarah J. Woodruff, Charles Boyer, Meghann Lloyd, Mark S. Tremblay

**Affiliations:** 1 Healthy Active Living and Obesity Research Group, Children’s Hospital of Eastern Ontario Research Institute, 401 Smyth Road, RI#1-214, Ottawa, ON K1H 8L1 Canada; 2 Faculty of Medicine, Department of Paediatrics, University of Ottawa, Ottawa, ON K1H 8M5 Canada; 3 Faculty of Human Kinetics, Department of Kinesiology, University of Windsor, Windsor, ON N9B 3P4 Canada; 4 Faculty of Health Sciences, University of Ontario Institute of Technology, Oshawa, ON L1H 7K4 Canada

**Keywords:** Assessment, Physical activity, Understanding, Instrument development, Psychometrics

## Abstract

**Background:**

Physical literacy is defined as the motivation, confidence, physical competence, and knowledge and understanding to engage in physical activity for life. Physical literacy knowledge and understanding encompasses movement (how to move), performance (evaluation of movement), and health and fitness (value of exercise, need for relaxation and sleep, etc.). This paper describes the development and evaluation of a standardized assessment of physical literacy knowledge and understanding for Canadian children in grades 4, 5, and 6.

**Methods:**

Proposed Physical Literacy Knowledge Questionnaire (PLKQ) content was identified through expert consultation and a review of provincial/territorial physical education curricula for grades 4 to 6. Open-ended questions verified language and generated response options. Feasibility was assessed via completion time and error frequency. Item validity assessed scores by age, gender, and teacher ratings of student knowledge. Test-retest reliability was assessed over short (2-day) and long (7-day) intervals.

**Results:**

Subsets of 678 children (54% girls, 10.1 ± 1.0 years of age) completed the feasibility and validity assessments. Response errors (missing or duplicate responses, etc.) were minimal (2% or less) except for one question (7% error) about the use of safety gear during physical activity. A Delphi process among experts in children’s physical activity and fitness achieved consensus on the core content and supported an item analysis to finalize item selection. As expected, knowledge scores increased with age (partial eta^2^ = 0.07) but were not related to gender (*p* = 0.63). Teacher ratings of children’s knowledge of physical activity behaviour (*r* = 0.13, *p* = 0.01) and fitness (*r* = 0.12, *p* = 0.03), but not movement skill (*r* = 0.07, *p* = 0.19) were associated with PLKQ scores. Test-retest reliability for PLKQ score and individual questions was substantial to excellent for 71% of comparisons over a 2-day interval, but lower over a 7-day interval (53% substantial or excellent). Items with low reliability had high or low proportions of correct responses.

**Conclusions:**

This study provides feasibility and validity evidence for the Physical Literacy Knowledge Questionnaire as an assessment of physical literacy knowledge for Canadian children in grades 4, 5, and 6. Completion rates were high and knowledge scores increased with age. Streamlining of the content in accordance with Delphi panel recommendations would further enhance feasibility, but would also focus the content on items with limited reliability. Future studies of alternative item wording and responses are recommended to enhance test-retest reliability.

**Electronic supplementary material:**

The online version of this article (10.1186/s12889-018-5890-y) contains supplementary material, which is available to authorized users.

## Background

Physical literacy is defined as the motivation, confidence, physical competence, and knowledge and understanding to engage in physical activity for life [1]. It is expected that children who have progressed further along their physical literacy journey are better able to adopt a healthy active lifestyle. It is also desirable that children with lower physical literacy be identified in order to provide them with additional support. According to Whitehead [2], physical activity motivation reflects a “willingness and eagerness” to take action that is demonstrated as a joy of movement, confidence in one’s own physical abilities, a positive attitude toward participation, and an expectation of successful participation. Physical competence reflects not only competence in movement skill but also the capacity (e.g., strength, endurance, etc.) for movement. Knowledge and understanding encompasses movement (how to move), performance (evaluation of movement), as well as health and fitness (value of exercise, need for relaxation and sleep, etc.). For brevity, we will use the term “knowledge” throughout this paper to represent the knowledge and understanding domain of physical literacy. Standardized protocols are available to assess the elements of motivation and confidence [3, 4], physical competence [5–8] and engagement in physical activity [9] in some age groups. However, although physical activity knowledge is a universally stated outcome of Canadian physical education curricula [10], a standardized measure of physical literacy knowledge and understanding has not been identified.

The purpose of this study was to develop and evaluate a standardized assessment of physical literacy knowledge and understanding. The target population was Canadian children in grades 4, 5, and 6 [11]. This pre-adolescent age group was selected because they have not yet experienced the decline in physical activity that occurs during adolescence [12], but are still able to independently respond to survey questions. The goal was to include an assessment of knowledge and understanding within the Canadian Assessment of Physical Literacy (CAPL) to ensure that the four domains of the CAPL (Knowledge and Understanding, Physical Competence, Motivation and Confidence, and Daily Behaviour) would be consistent with the current Canadian consensus definition of physical literacy [1].

## Methods

### Study design overview

**Fig. 1:**
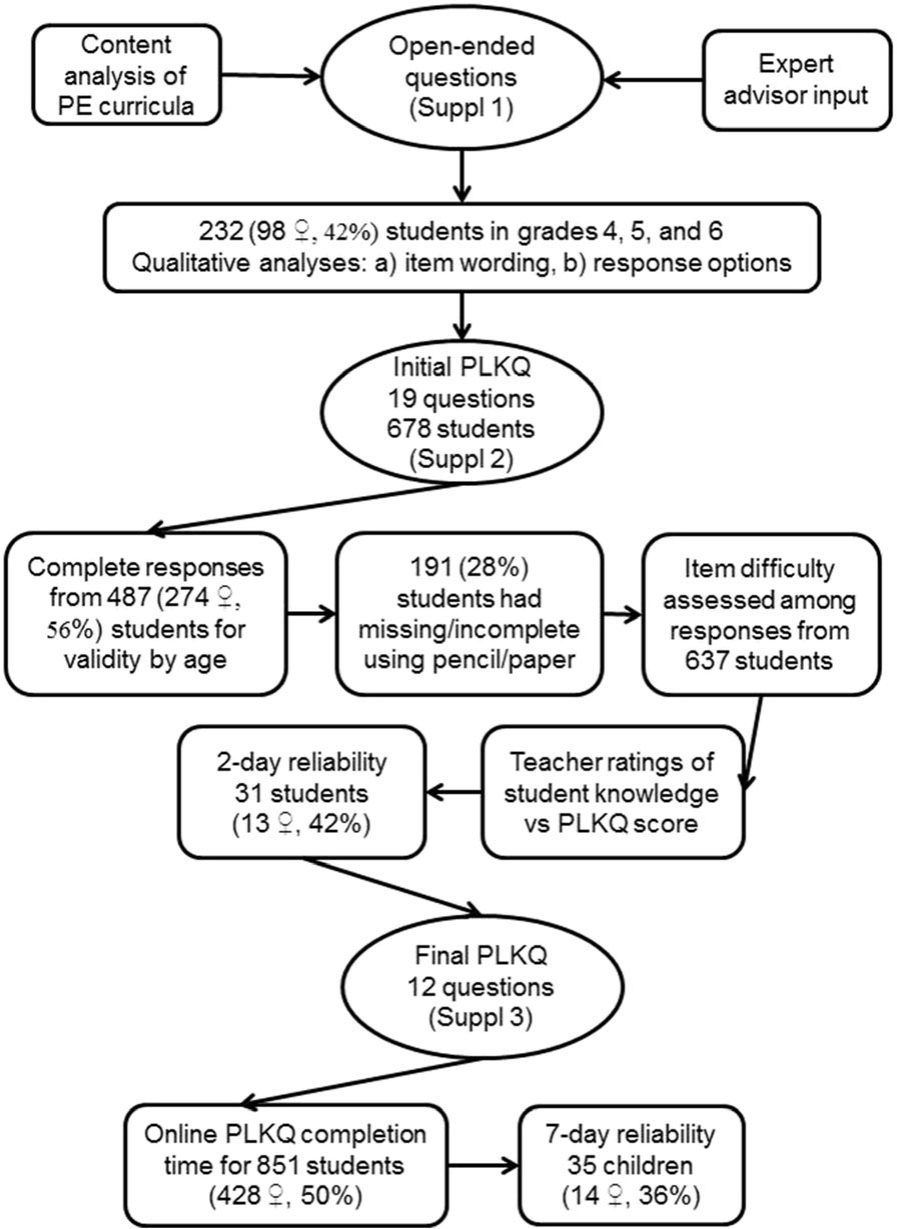
Overview of research to develop the Physical Literacy Knowledge Questionnaire*. * All participants in all phases of this research described above were students in grades 4, 5, or 6, or children attending summer camps who would be in grades 4, 5, or 6 when returning to school after summer vacation

The development of the Physical Literacy Knowledge Questionnaire (PLKQ) was completed through a series of studies as described in Fig. 1. Initially, proposed content was identified through a review of physical literacy knowledge components of the physical and health education curricula, combined with input from education professionals and expert advisors. Potential questions (Additional file 1) were then tested by providing students in grades 4, 5, and 6 with the opportunity to respond in an open-ended format. Responses were qualitatively analyzed to optimize item wording and to generate a list of response options suitable for a closed-ended format. Feasibility of the initial PLKQ (Additional file 2) was evaluated by having students respond, in pencil and paper format, to the closed-ended questions. Response errors and teacher reports of each student’s knowledge were evaluated. Reliability was assessed by having students complete the PLKQ on two separate occasions. The datasets supporting the conclusions of this article are available from Dr. Patricia Longmuir.

### Participants

Study participants were convenience samples of children attending schools and summer camps in Ontario, Canada, who had agreed to cooperate with our research. All children at collaborating schools and camps were approached to participate, and those who assented and whose parents provided written consent were enrolled. Children tested in schools were in grades 4, 5, or 6. Children tested in summer camps were going to be in grades 4, 5, or 6 when they returned to school in September. The majority of study participants were aged 9 (Grade 4), 10 (Grade 5) or 11 (Grade 6) years. There were smaller numbers of children 8 and 12 years of age. Children 8 years of age were in Grade 4 and born late in the year (October/November/December) but who were tested in the fall (September to December) before their ninth birthday. Children 12 years of age were in Grade 6 and born early in the year (January/February/March/April) and tested after their 12th birthday.

Informed written consent was obtained from the parents of all children before enrolment. Teachers who participated in the rating of student knowledge also provided written informed consent prior to study participation. Verbal child assent was obtained before the commencement of study activities. Study activities were approved by the Children’s Hospital of Eastern Ontario and University of Windsor Research Ethics Boards as well as by the research committees of participating school boards and camps.

### Process of development for the PLKQ

Content areas for the PLKQ were systematically identified through a review of physical and health education curricula from all Canadian provinces and territories, supplemented by the recommendations of an international Delphi process [13]. Key learning objectives from each curriculum document for grades 4, 5, or 6 were identified (Table 1). A content analysis of the key learning objectives identified the following areas of knowledge as being common across all of the reviewed curricula: importance of physical activity, definition of cardiorespiratory fitness, guidelines for daily physical activity and sedentary time, definition of “healthy”, recognition of movement skills, understanding of fitness and its impact on physical activity, safety practices during physical activity, identification of healthy foods, and methods of skill and fitness improvement. Open-ended questions on the proposed topics to be assessed were then provided to children in grades 4, 5, and 6 as well as their teachers (Additional file 1). Feedback was obtained on the clarity and wording of the questions from both teachers and students. In addition, the children’s responses to the open-ended questions were used to identify the closed-ended response options that would be included in the initial PLKQ (Additional file 2).

**Table 1:** Overview of physical and health education curriculum learning objectives

Knowledge content	B.C./Yukon			Alberta/N.W.T.			Saskatchewan			Manitoba/Nunavut			Ontario			Quebec			New Brunswick			Nova Scotia			P.E. I.			Newfoundland/Labrador		
	Gr. 4	Gr. 5	Gr. 6	Gr. 4	Gr. 5	Gr. 6	Gr. 4	Gr. 5	Gr. 6	Gr. 4	Gr. 5	Gr. 6	Gr. 4	Gr. 5	Gr. 6	Gr. 4	Gr. 5	Gr. 6	Gr. 4	Gr. 5	Gr. 6	Gr. 4	Gr. 5	Gr. 6	Gr. 4	Gr. 5	Gr. 6	Gr. 4	Gr. 5	Gr. 6
1. Like playing sports																			**√**	**√**	**√**				**√**	**√**	**√**			
2. 3 things like about sports																			**√**	**√**	**√**	**√**			**√**	**√**	**√**			
3. 3 things dislike about sports																						**√**								
4. Name types of exercise				**√**	**√**	**√**			**√**			**√**				**√**	**√**	**√**	**√**	**√**	**√**	**√**	**√**	**√**						
5. Importance/exercise benefits	**√**	**√**	**√**				**√**	**√**	**√**		**√**	**√**	**√**	**√**					**√**	**√**	**√**	**√**	**√**	**√**	**√**	**√**	**√**	**√**	**√**	**√**
6. How much exercise/day	**√**	**√**	**√**										**√**	**√**	**√**						**√**	**√**	**√**	**√**						
7. Girl walking or running																														
8. Types of physical fitness	**√**	**√**	**√**	**√**	**√**	**√**	**√**	**√**	**√**	**√**	**√**		**√**	**√**					**√**	**√**	**√**	**√**	**√**	**√**						
9. Importance of healthy food	**√**	**√**	**√**	**√**	**√**	**√**	**√**	**√**	**√**	**√**		**√**	**√**	**√**	**√**				**√**	**√**	**√**	**√**	**√**	**√**						
10. Why do you play sports																			**√**	**√**										
11. How long to get to school																														
12. Reasons not to participate									**√**												**√**	**√**			**√**	**√**	**√**			
13. Better thrower													**√**																	
14. All sports you play																														
15. Do your friends exercise																														
16. Do you play outside													**√**										**√**	**√**						
17. Jogging 20 min. Improves													**√**																	
18a. Being fit	**√**	**√**	**√**	**√**	**√**	**√**	**√**	**√**	**√**	**√**	**√**		**√**	**√**	**√**				**√**	**√**	**√**		**√**		**√**	**√**	**√**			
18b. Daily activity	**√**	**√**	**√**	**√**	**√**	**√**	**√**	**√**	**√**		**√**	**√**	**√**	**√**	**√**						**√**									
18c. Physical activity	**√**	**√**	**√**				**√**	**√**	**√**			**√**	**√**	**√**	**√**						**√**									
18d. Increase in exercise			**√**	**√**	**√**	**√**	**√**	**√**	**√**			**√**	**√**	**√**	**√**															
18e. Heart rate	**√**		**√**						**√**	**√**	**√**	**√**	**√**	**√**	**√**															
19. How much T.V.																														
20. How much time on computer																														
21. Motor skills are important													**√**								**√**									

### Feasibility of the PLKQ

Feasibility analyses were conducted among children assessed at schools in eastern Ontario during development of the CAPL. Children completed the initial PLKQ (Additional file 2) during class time. The proportion of children in grades 4, 5, and 6 able to complete the initial PLKQ without missing or other completion errors (e.g., multiple responses to one item) was evaluated. Completion time was analyzed in relation to instrument feasibility. A factor analysis assessed the difficulty of each question based on the proportion of correct/incorrect responses.

### Validity of the PLKQ

Content for the initial PLKQ was verified through an international Delphi process [13]. Experts in children’s physical activity, movement, motivation, and fitness achieved consensus on initial PLKQ content through an iterative process. Results from administration of the initial PLKQ to children assessed in schools in eastern Ontario were analyzed by the child’s self-reported age and gender. It was hypothesized that knowledge would not vary by gender but would increase with age.

### Reliability of the PLKQ

Test-retest reliability data were collected from two samples of children who were asked to complete the PLKQ twice. One sample (*n* = 31) attended a summer camp in eastern Ontario, and completed the initial PLKQ on 2 separate days, at an interval of 2 days. A second sample of children (*n* = 35) completed the final PLKQ (Additonal file 3), a more streamlined version, at their school in southwestern Ontario, on 2 occasions over a 1-week interval. Test-retest reliability of the PLKQ responses was assessed using Pearson correlation coefficients.

### Statistical analyses

Descriptive statistics were used to summarize the data (mean ± SD, frequencies) and the participants in each phase of this research. Regression analyses investigated the impact of independent variables on study outcomes. Correlations were used to evaluate test-retest reliability. Due to the large sample size used for the feasibility and validity analyses, results were interpreted based on measures of effect size. According to Murphy and Myors [14], small, medium, and strong effects are observed when partial eta squared exceeds 0.01, 0.06, or 0.14, respectively. Correlations are defined as small (> 0.10), moderate (> 0.30), strong (> 0.50), substantial (> 0.70), or excellent (> 0.90).

## Results

### Feasibility of the PLKQ

Feasibility and validity analyses were conducted on the same sample of children, whose demographic information is summarized in Table 2. A total of 678 children (54% girls), mean age 10.1 ± 1.0 years completed the feasibility assessment of the initial PLKQ. Of these, a calculated PLKQ score was available for 487 children (28% had response errors [i.e., were missing or responded inappropriately to two or more items]). Among the 191 children with response errors, the number and type of errors for each item in the initial PLKQ are summarized in Table 3. A factor analysis demonstrated a good distribution of easy, moderate, and difficult questions (Table 4).

**Table 2:** Feasibility and validity study (initial PLKQ) participants by age, gender, and completion of Physical Literacy Knowledge Questionnaire

Age	All participants			Complete PLKQ score		
	Boys	Girls	Total	Boys	Girls	Total
8 years^a^	4	5	9	3	5	8
9 years	94	104	198	61	84	145
10 years	94	123	217	72	88	160
11 years	91	107	198	63	81	144
12 years^a^	27	29	56	14	16	30
Total	310	368	678	213	274	487

^a^ All children were in grades 4, 5, or 6. Children 8 years of age were in Grade 4 and born late in the year (October/November/December) who were tested in the fall before their ninth birthday. Children 12 years of age were in Grade 6 and born early in the year (January/February/March/April) and tested after their 12th birthday

*PLKQ* Physical Literacy Knowledge Questionnaire

**Table 3:** Frequency and type of errors in completion of Physical Literacy Knowledge Questionnaire

PLKQ item (see Additional file 1)	Incomplete or missing	Multiple answers	Limited comprehension	Child indicated question did not apply
How important is daily PA?	1	2	1	0
How important to be more active?	5	1	5	0
Reasons to be active?	8	**15**	1	0
Reasons not to be active?	7	8	0	0
Compared to peers, how active are you?	3	1	0	0
Compared to peers, how good at sports?	5	0	2	0
How many minutes of DPA at school?	2	0	0	1
How many minutes of PA daily?	1	0	0	1
Most time children should sit still?	3	2	0	0
Words that mean healthy?	1	0	1	0
What sport skill is shown?	1	0	2	0
Story about building fitness	6	0	0	0
Sunscreen wear time in summer?	5	0	0	0
Sunscreen wear time in winter?	6	0	0	1
Which are healthy foods?	9	3	**14**	2
Which PA do you wear safety gear?	8	0	**50**	0
Hours of sleep per day?	8	0	0	1
Parent support for your PA	**22**	2	1	2
Peer support for your PA	11	3	1	0
How to improve your skill?	4	1	0	0
How to get in better shape?	3	1	0	0
Active transport to school?	**20**	8	12	2
Active transport to school?	**23**	11	**17**	2
Choice to do after school?	6	**17**	1	1
How much time using a computer?	12	0	1	0
How much time for TV, etc.?	12	0	1	0

*DPA* daily physical activity, *PA* physical activity, *PLKQ* Physical Literacy Knowledge Questionnaire

Bold numbers indicate error rates exceeding 2% of responses

**Table 4:** Analysis of initial PLKQ question difficulty among 637 respondents

Question	Average score or % correct response	Measure	Difficulty^a^
1. Physical activity importance	8.65 ± 1.6 (out of 10)	−1.56 S.E. = 0.08	Easy
2. Importance to be more active than you are now	5.01 ± 2.7 (out of 10)	0.64 S.E = 0.05	Moderate
5. Compared to other kids how active are you?	6.91 ± 2.1 (out of 10)	− 0.76 S.E. = 0.06	Easy
6. Compared to other kids your age how good are you at sports or skills?	6.58 ± 2.39 (out of 10)	− 0.3 S.E. = 0.06	Moderate
7. Cardiorespiratory means?	48% (300/626)**	0.44 S.E. = 0.08	Moderate
8. MVPA at school	11.9% (79/665)	2.44 S.E. = 0.12	Hard
9. MVPA for the whole day?	70% (441/632)**	− 0.42 S.E. = 0.08	Moderate
10. Minutes of sedentary time per day?	19% (122/635)	1.78 S.E. = 0.09	Hard
11. Healthy means? (total score = 11)	4.91 ± 1.8 (out of 11)	0.22 S.E. = 0.09	Moderate
12. Identify sport skill	68.8% (451/656)	−0.47 S.E. = 0.08	Moderate
13. Fill in the spaces in the story	5.96 ± 2.0** (out of 9)	−0.67 S.E. = 0.11	Moderate
14. Sunscreen in the summer	62% (376/606)	−0.07 S.E. = 0.08	Moderate
15. Sunscreen in the winter	3.4% (19/606)	3.75 S.E. = 0.22	Hard
16. Identify healthy foods	2.81 ± 0.49 (out of 3)	− 1.33 S.E. = 0.08	Easy
17. Wearing safety equipment	2.77 ± 3.5*, ** (out of 11)	–	–
21. Skill acquisition	30% (189/436)	1.09 S.E. = 0.08	Hard
22. How to get in better shape	88% (551/632)	−1.52 S.E. = 0.11	Easy
25. Preferred activities after school	68% (413/608)	–	–
26. Sedentary time spent on computer	6.61 ± 2.5 (out of 15)	− 0.13 S.E. = 0.04	Moderate
27. Sedentary time spent watching TV	7.81 ± 2.4 (out of 15)	0.15 S.E. = 0.04	Moderate

*gender difference

**age difference

^a^Difficulty of each measure was assessed by using the following criteria: < − 0.7 = easy; − 0.7 to 0.7 = moderate; > 0.7 = hard

*MVPA* moderate to vigorous physical activity, *S.E.* standard error

The most common response error occurred with the question regarding safety gear. Children were presented with pictures of different types of physical activity (see question #16, Additional file 2). They were asked to circle the activities that they themselves perform, and then add a check mark to the activities for which they wear safety gear (e.g., helmet, elbow pads). The score assigned was based on the proportion of correct responses among only the activities that the child actually performs. Fifty (7%) children showed a limited comprehension of the question instructions, as they checked pictures that they had not circled, indicating that they knew safety gear was required but did not understand the instruction to circle the activities that they actually performed. Other common errors, each affecting about 2% of responses, were missing information or lack of understanding for the questions regarding whether the child uses active transportation to get to school, and multiple answers in response to questions about activity preferences after school and reasons for being active.

Mean completion time was assessed in order to further clarify the feasibility of the PLKQ. Among 117 children who were timed completing the PLKQ using the pen and paper format, the mean completion time was 43, 42, and 38 min for students in grades 4, 5, and 6, respectively. Among 851 children who completed the PLKQ using the online platform (www.capl-ecsfp.ca), completion time was 27 min for Grade 4 students, 26 min for Grade 5 students, and 14 min for students in Grade 6. These analytics track only the time that each student required to complete the knowledge component of the PLKQ, which is comprised of 9 questions requiring 38 responses. The time required for questions #2, #3, and #12 of the PLKQ, which are part of the Motivation and Confidence domain score, were excluded.

### Validity of the PLKQ

Table 5 summarizes the consensus recommendations from the Delphi panel with regard to the PLKQ content. The Delphi expert panel (*n* = 19, 4 female [21%]), who had 25 ± 15 years of research experience within their field (range: 5 to 65 years) and a combined total of 4181 peer-reviewed publications (range 15 to 1500), agreed that children’s knowledge of daily physical activity and screen time guidelines, the meaning of cardiorespiratory fitness and muscular strength and endurance, and how to improve sport skills and fitness were important areas of knowledge that should be included in the PLKQ. There was also consensus for the inclusion of questions about the child’s use of active transportation and self-reported days per week of moderate to vigorous physical activity. Questions probing the child’s understanding of the meaning of “healthy” and the need for safety gear during physical activity were also close to consensus (70% and 72% respectively, where 75% was required for consensus). Other areas of consensus among the Delphi panel participants were that factor item analysis should be used to determine the final content for the PLKQ (see paper by Gunnell et al. in this issue), and that there was a need to ensure those administering the CAPL had the ability to calculate a Knowledge and Understanding domain score.

**Table 5:** Summary of Physical Literacy Knowledge Questionnaire recommendations from Delphi Panel of 19 international experts

The Knowledge and Understanding domain should include:	Strongly agree	Agree	Neutral	Disagree	Strongly disagree	Consensus
Self-reported sedentary time	12%	35%	12%	24%	18%	No
Self-reported sleep time	12%	29%	6%	29%	24%	No
**Knowledge of daily MVPA guidelines**	53%	35%	6%	0%	0%	**Yes**
**Knowledge of screen time guidelines**	35%	53%	12%	0%	0%	**Yes**
**The meaning of cardiorespiratory fitness**	35%	59%	0%	6%	0%	**Yes**
**The meaning of muscular strength/endurance**	29%	53%	6%	6%	6%	**Yes**
The meaning of “healthy”	29%	41%	29%	0%	0%	No
Ability to identify the skill being performed	18%	18%	59%	6%	0%	No
Understanding the benefits of PA (missing words question)	1%	41%	41%	0%	0%	No
Summer sunscreen use	0%	18%	41%	12%	29%	No
Winter sunscreen use	0%	6%	47%	18%	29%	No
Safety gear use	6%	65%	12%	18%	0%	No
**How to get better at a sport skill**	47%	41%	12%	0%	0%	**Yes**
**How to get in better shape**	35%	59%	6%	0%	0%	**Yes**
**Item analysis should be conducted on each question to determine which questions are retained in the final questionnaire**	37%	53%	11%	0%	0%	**Yes**
Question 16 (indicate which foods are healthy/unhealthy) should not be included as it assesses diet rather than physical literacy	11%	47%	26%	11%	5%	No
**A question that assesses the child’s knowledge relative to strength and muscular endurance should be added**	16%	79%	5%	0%	0%	**Yes**
**When assessing physical literacy it is important to include a question about the child’s use of active modes of transportation**	21%	68%	5%	0%	5%	**Yes**
Cognition, attitudes, and efficacy measures should be norm referenced	5%	21%	26%	47%	0%	No
**A question regarding the number of days per week that children participate in MVPA should be added to the CAPL protocol**	32%	63%	5%	0%	0%	**Yes**
**The option to calculate a Knowledge and Understanding domain composite score should be provided**	41%	41%	18%	0%	0%	**Yes**
Each Knowledge and Understanding question should be assigned equal weighting when calculating an overall activity knowledge score	29%	29%	6%	29%	6%	No

Note: Open-ended Round #1 responses had consensus that a physical activity knowledge assessment should be included in the Canadian Assessment of Physical Literacy. **Bold** items achieved consensus among Delphi participants

*CAPL* Canadian Assessment of Physical Literacy, *MVPA* moderate to vigorous physical activity, *PA* physical activity

In a linear regression model, knowledge of physical literacy as assessed by the initial PLKQ was significantly associated with increased age (*F* = 174.5, *p* < 0.001, partial eta squared = 0.068 (medium effect [15]); Table 6). There was no relationship between initial PLKQ score and self-reported gender (*F* = 0.24, *p* = 0.63).

**Table 6:** Physical Literacy Knowledge Questionnaire score by age

Self-reported age^a^	n (% girls)	Average knowledge score^b^ (range)
8 years	8 (63%)	10.03 ± 1.5 (8 to 13)
9 years	145 (58%)	9.91 ± 2.3 (5 to 17)
10 years	160 (55%)	10.39 ± 2.5 (1 to 15)
11 years	144 (56%)	10.89 ± 2.5 (4 to 16)
12 years	30 (53%)	11.01 ± 2.5 (5 to 16)

^a^Age was significantly associated with initial PLKQ score (*F* = 174.5, *p* < 0.001)

^b^Maximum score for the initial PLKQ was 18 points

Table 7 compares teacher ratings of the children’s knowledge about physical activity behaviour, fitness, and movement skill to the children’s initial PLKQ score. Teachers were asked to rate each child’s knowledge on a scale from 1 (low knowledge) to 10 (excellent knowledge). Separate ratings were provided for knowledge of physical activity behaviour, physical fitness, and movement skill. Teachers provided ratings for those students to whom they taught physical education classes. Twenty-five teachers (5 [20%] male) rated 516 students (median 23 students per teacher, range 2 to 37). Significant correlations were observed between the initial PLKQ score and teacher ratings of the child’s knowledge of physical activity behaviour and fitness. Teacher ratings of the child’s knowledge about movement skill were not associated with the initial PLKQ score. In a multi-variable regression model adjusted for the child’s self-reported age and gender, only the teacher rating of the child’s fitness knowledge was significantly associated with initial PLKQ score (*F* = 4.3, *p* = 0.005, partial eta^2^ = 0.034 [small effect]). For each 1-year increase in self-reported age, the initial PLKQ score increased by 0.36 points. For each 1-point increase in the teacher rating of the child’s fitness knowledge, the initial PLKQ score increased by 0.19 points. Gender was not significantly related to PLKQ score (*p* = 0.71).

**Table 7:** Association between Physical Literacy Knowledge Questionnaire score and teacher ratings of a child’s knowledge

	Initial PLKQ score	Teacher rating of behaviour knowledge	Teacher rating of fitness knowledge	Teacher rating of skills knowledge
Initial PLKQ score	1.0 (*p* < 0.001) *n* = 487	–	–	–
Teacher rating of child’s knowledge of physical literacy behaviour	0.13 (*p* = 0.011) *n* = 366	1.0 (*p* < 0.001) n = 514	–	–
Teacher rating of child’s knowledge of fitness	0.12 (*p* = 0.027) *n* = 366	0.69** (*p* < 0.001) *n* = 514	1.0 (*p* < 0.001) n = 514	–
Teacher rating of child’s knowledge of movement skill	0.07 (*p* = 0.192) *n* = 364	0.67** (*p* < 0.001) *n* = 512	0.89** (*p* < 0.001) n = 512	1.0 (*p* < 0.001) n = 512

** strong correlation

### Reliability of the PLKQ

The self-reported age and gender for the children who participated in the reliability evaluation of the PLKQ are provided in Table 8. Thirty-one children (13 [42%] girls, mean age 10.6 ± 1.3 years) completed the test-retest reliability assessment of the initial PLKQ over a 2-day interval. These children completed the initial PLKQ during the lunch break at their summer day camp. The day camp offered a variety of sports and camp activities, and was hosted at a local university. A second sample of 35 children (14 [36%] girls, mean age 9.8 ± 0.7 years) completed the test-retest reliability assessment over a 7-day interval while at school. This second sample of children completed the final, shorter version of the PLKQ (Additional file 3).

**Table 8:** Demographic information for children completing the Physical Literacy Knowledge Questionnaire reliability assessment

Age	2-day interval			7-day interval		
	Boys	Girls	Total	Boys	Girls	Total
8 years^a^	0	3	3	0	0	0
9 years	1	1	2	5	7	12
10 years	4	4	8	12	6	18
11 years	6	3	9	4	1	5
12 years^a^	7	2	9	0	0	0
Total	18	13	31	21	14	35

^a^ All children were in grades 4, 5, or 6. Children 8 years of age were in Grade 4 and born late in the year (October/November/December) who were tested in the fall before their ninth birthday. Children 12 years of age were in Grade 6 and born early in the year (January/February/March/April) and tested after their 12th birthday

The test-retest correlation coefficients for the PLKQ total score, as well as for individual items on the PLKQ, are provided in Table 9. Reliability of the PLKQ total score was strong (*r* = 0.62 and 0.69 over the 2- and 7-day intervals, respectively). Adjusting the correlation by age did not alter the reliability (*r* = 0.60 and 0.70 over the 2- and 7-day intervals, respectively). Over a 2-day interval, the reliability of most individual items was substantial to excellent. Responses to questions about the meaning of “cardiorespiratory fitness”, a story about sport training and fitness, and how to get in better shape had moderate reliability. Item reliability over a 7-day interval was similar for most questions. Reliability was lower over a 7-day interval, compared to the 2-day interval, for questions asking about the use of safety gear during physical activity and the meaning of “healthy”.

**Table 9:** Test-retest reliability of the Physical Literacy Knowledge Questionnaire over 2- and 7-day intervals

	2-day interval	7-day interval
**PLKQ total score**	**Correlation** ^a^ **(n)**	**Correlation** ^a^ **(n)**
PLKQ total score	0.62 (31)	0.69 (35)
**Individual PLKQ questions**	**Correlation** ^a^ **(n)**	**Correlation** ^a^ **(n)**
How important do you think it is that you are physically active every day?	**0.80 (31)**	Not asked
How important is it to you to be more active than you are now?	**0.71 (30)**	Not asked
**Compared to other kids your age, how active are you?**	**0.69 (31)**	**0.86 (35)**
**Compared to other kids your age, how good are you at sports or skills?**	**0.84 (31)**	0.64 (35)
Cardiorespiratory means…	0.52 (30)	0.54 (35)
Musculoskeletal fitness means …	Not asked	0.25 (35)
**Minutes of moderate or vigorous physical activity every day at school?**	**0.80 (31)**	Not asked
Total minutes of moderate or vigorous physical activity daily?	0.20 (30)	0.61 (35)
What is the most time that children should sit still each day?	0.33 (30)	0.34 (35)
**Healthy means …**	**0.84 (31)**	0.60 (35)
Which sport skill are they doing?	0.63 (30)	Not asked
Fill in the blanks to create a story about sport training and fitness	0.48 (30)	0.59 (35)
In the summer, how often do you wear sunscreen?	1.00 (31)	Not asked
In the winter, how often do you wear sunscreen?	No correct answers	Not asked
**Which foods are healthy to eat?**	**0.87 (25)**	Not asked
**How many hours do you usually spend sleeping each day?**	**0.86 (31)**	Not asked
**Tell us which activities you do and whether or not you wear safety gear?**	**0.87 (31)**	0.45 (35)
If you wanted to get better at a sport skill, what should you do?	0.62 (31)	0.60 (35)
If you wanted to get in better shape, what would be the best thing to do?	0.47 (31)	0.49 (35)
**If you could choose what you did after school, what would you pick?**	**0.86 (31)**	**0.93 (35)**

^a^ Correlations were defined as small (> 0.10), moderate (> 0.30), strong (> 0.50), substantial (> 0.70), or excellent (> 0.90), per Murphy and Myors [14]. Substantial or excellent correlations are shown in **bold** text

*PLKQ* Physical Literacy Knowledge Questionnaire

Reliability for the question that asked children about the recommended amount of daily sedentary time was moderate over both the 2- and 7-day intervals (*r* = 0.33 and 0.34, respectively). Even though the proportion of children answering correctly was similar (21 and 20%, respectively), a paired comparison of responses was significantly different (*p* = 0.045) between the first and second trials over the 7-day interval. Over a 7-day interval, 72% and 77% of children correctly identified the recommended daily physical activity (1st and 2nd trial, respectively) and the correlation was strong (*r* = 0.61). Over a 2-day interval, reliability was low (0.20) and may have been influenced by the content of the initial PLKQ, which asked separately about moderate to vigorous activity at school and total throughout the day.

## Discussion

Generally, the PLKQ proved to be feasible, valid, and reliable for children in grades 4, 5, and 6 (8 to 12 years of age). Rates for missing or incomplete responses, assessed using the pen and paper format of the initial PLKQ, were low. Less than 2% of all questions had incomplete or missing responses, except for the question asking about safety gear, for which 50 children indicated the need for safety gear without indicating that they participated in the activity. Use of the online format for the PLKQ requires complete responses in order to log off from the website. Validity of question content was supported through a Delphi process, a balanced array of item difficulty, and the finding that PLKQ scores increased with age but did not differ by gender. Test-retest reliability was substantial to excellent for most questions over a 2-day interval, with some questions having moderate reliability, particularly over a 7-day interval.

The Delphi panel achieved consensus for the inclusion of 6 content areas within the PLKQ: knowledge of daily physical activity and screen time guidelines, the meaning of cardiorespiratory fitness and muscular strength/endurance, and how to improve fitness or sport skills [13]. Content related to the use of safety gear and the meaning of “healthy” came very close to achieving consensus. The Delphi panel was either neutral or in agreement with content items for the benefits of sport and fitness participation and the identification of movement skills. These items were retained in the PLKQ to be inclusive of relevant theoretical concepts and to more fully reflect the physical and health education curricula. The Delphi panel was either neutral to, or disagreed with, the inclusion of item content related to self-reported sleep time and the use of sunscreen; these items were subsequently removed when the streamlined final PLKQ was created. The inclusion of self-reported sedentary time was supported by 47% of the Delphi panel, but opposed by 42% of those experts. Those opposed indicated that self-reported sedentary time was a measure of behaviour rather than knowledge. Therefore, the item was removed from the final PLKQ.

Teacher ratings of each child’s knowledge of fitness, physical activity behaviour, and movement skill were intended to contribute to the assessment of PLKQ validity. Although there were statistically significant (*p* < 0.05) correlations between PLKQ total score and teacher ratings of children’s physical activity and fitness knowledge, the correlations were small in magnitude (*r* < 0.15) and, therefore, explained less than 2% of the variance in initial PLKQ responses. PLKQ total score was not significantly correlated with teacher ratings of children’s knowledge of movement skill. If teacher ratings of the children’s knowledge were assumed to be the “gold standard” reference, these results would seem to suggest that the initial PLKQ may have limited validity as an assessment of physical literacy knowledge. However, the three teacher ratings by content area (fitness, behaviour, movement skill) were strongly associated (*p* > 0.60) with each other, suggesting that teachers had consistent expectations for physical literacy knowledge, such that students were rated as having either good or poor knowledge in all content areas. Teacher ratings were provided by each child’s physical education teacher. At times this was the regular classroom teacher and at other times it was someone else (e.g., physical education specialist, camp or program leader). Therefore, the limited association between teacher ratings and initial PLKQ scores may reflect the fact that some teachers had no knowledge of the child’s classroom learning or performance. Future research should evaluate the relationship between student knowledge and teacher ratings, adjusting for teacher familiarity with the student’s classroom performance. These results may also reflect the ability of the initial PLKQ to discriminate between the child’s knowledge in different physical literacy content areas.

While test-retest reliability for most items (71%) on the initial PLKQ was substantial to excellent over a 2-day interval, the reliability of items on the final PLKQ was lower over a 7-day interval (53% substantial or excellent). Items with limited reliability over both short and longer intervals included daily recommendations for physical activity and screen time, benefits of sport training and fitness, and how to “get in better shape”. It is possible that differences in reliability of these items occurred because children were curious about the correct answer to these questions after the first trial, and were prompted to seek out the correct response prior to the second trial. Data supporting this explanation come from the question about recommended daily physical activity. Over a 2-day interval, only 4 children had an incorrect response on the first day of testing, and 3 of these 4 answered the question correctly on the second day. Over the 7-day interval, only 9 of 35 responses were incorrect on the first day, with 3 becoming a correct response when the assessment was repeated. In spite of the limited reliability for these items, they were retained in the final PLKQ based on the Delphi panel consensus support and theoretical arguments that this knowledge is important to evaluate.

Low reliability for the question asking how to “get in better shape” may have reflected different interpretations. Examiners reported that some children asked if the question meant to improve one’s physical appearance. Although “get in better shape” was the preferred wording identified in response to the initial open-ended questions (Additional file 1), a change to the wording of this item may improve understanding of the question’s intent, ultimately improving the reliability of responses. Responses to the items relating to daily screen time recommendations and the benefits of fitness and sport training suggest that only a small proportion of children could respond correctly. The screen time item has 4 response options (1 correct and 3 incorrect). The “benefits of fitness and sport training” item was a paragraph / story that required children to fill in 9 (initial PLKQ) or 5 (final PLKQ) blank spaces from a list of word options provided. If most children did not know the correct responses to these items, and therefore were “guessing”, it is reasonable to expect that the lower reliability measures for these questions may reflect the children’s need to randomly choose among multiple response options.

The mean time required to complete the final PLKQ was estimated to be 14 min for students in Grade 6, but 27 min for students in grades 4 and 5. This represents a substantial time burden for the younger students, particularly if there is also a desire to assess their physical literacy motivation. Future research is needed to reduce the response burden for younger students.

### Strengths and limitations

To our knowledge, this study is the first report of the psychometric properties of a physical literacy knowledge assessment for children. Feasibility was assessed in a large sample of Ontario children attending grades 4, 5, and 6 classes. The content of the assessment was matched to the physical and health education curricula published for each Canadian province and territory, and verified through a Delphi expert consensus process [13]. Participants in all phases of this research were convenience samples of children attending local schools or day camps in Ontario. Therefore, the extent to which the study sample is representative of the population of Canadian children as a whole remains unknown. Additional investigations of item reliability are recommended given the relatively small sample sizes reported here, and differences in the item content between the 2- (initial PLKQ) and 7-day (final PLKQ) intervals. The psychometric properties of questions modified in the future should also be assessed and reported.

## Conclusions

The results of this study provide evidence for the feasibility, reliability, and validity of the PLKQ as an assessment of physical literacy knowledge and understanding among Canadian children in grades 4, 5, and 6. Completion rates were high and knowledge scores increased with age. Streamlining of the content in accordance with Delphi panel recommendations would further enhance feasibility, but would also focus the content on items with lower reliability. Future studies of alternative item wording and responses are recommended to enhance test-retest reliability.

## Additional files

Additional file 1: Original questionnaire with open-ended questions (as of 2009-09-02). (PDF 662 kb)

Additional file 2: Version 1 of the Cognitive domain questions (as of 2012-02-14). (DOCX 1747 kb)

Additional file 3: Final version of the Physical Activity knowledge questions (as of 2013-06-28). (DOC 236 kb)

## Abbreviations

CAPL: Canadian Assessment of Physical Literacy
PLKQ: Physical Literacy Knowledge Questionnaire
