# Supplementary material for: Physical Literacy Knowledge Questionnaire: feasibility, validity, and reliability for Canadian children aged 8 to 12 years
Source: BMC Public Health. 2018 Oct 2;18(Suppl 2):1035. doi: 10.1186/s12889-018-5890-y (PMC6167766; doi:10.1186/s12889-018-5890-y)
Supplement: Supplementary file 1 — Original questionnaire with open-ended questions (as of 2009-09-02). (PDF 662 kb) [file 12889_2018_5890_MOESM1_ESM.pdf]

Physical Education Knowledge

(Canadian Assessment of Physical Literacy)

Name: ______________________________ Age: ___________

I am in grade: 4 5 6 (please circle one)

I am a: boy girl (please circle one)

In this project, when we talk about physical activity, we mean things that you do for at least 20 minutes that make you breathe harder.

Why are we asking you these questions? We want to know what kids like you know about physical activity, sports and exercise.

Please remember:

- **There are no right or wrong answers. If you do not know the answer, please give your best guess.**
- **There is no time limit, so please take all the time you need.**
- **Please use the space under each question to write your answer.**

CHEO Research Ethics Board
APPROVAL
Chair's Signature:
Date:

1. Name **3** things you <u>like</u> about playing sports or being physically active.

   ____

   ____

   ____

2. Name **3** things you **<u>don't like</u>** about playing sports or being physically active.

   ____

   ____

   ____

ID #

3. Running, throwing, jumping, catching and kicking are skills we use all the time in physical activities. **Why do you think that they are important?**

____

4. **Cardiorespiratory fitness is… (circle the right answer)**
   a) The muscle's ability to contract.
   b) The hearts ability to pump blood and the lungs ability to provide oxygen.
   c) Our heart rate.
   d) Our ability to run and do sports that we like.

5. **Do you think it is important to be physically fit?** **Yes** **No**
(please circle one)

Why? ____

6. **How long should you and other Canadian children engage in physical activity every day?**

_______ minutes.

7. **Can you name 2 reasons why some children don't/can't participate in sports or physical activity?**
   1. ____
   2. ____

8. **Draw a line to all the words you think describes what "Healthy" is.**

Being free from disease

Exercising

looking good

Good nutrition

feeling good

Healthy is...

Being skinny

Being flexible

Being attractive

Being happy

Strong muscles

Having endurance

Being popular

ID #

9. All of the athletes in the photos below are doing the same action.
Which sport skill are they doing? ____________________

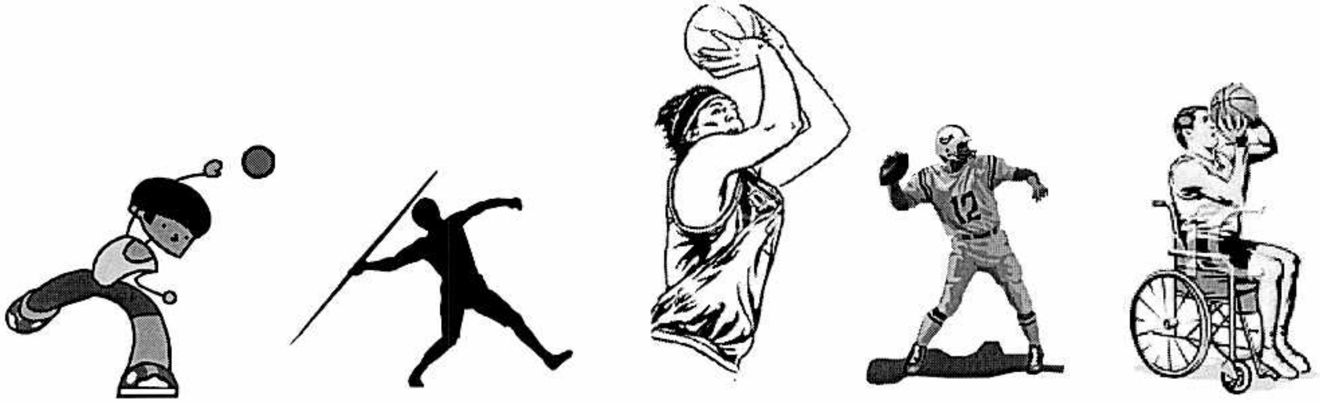

10. When you are **not** at school what sports or activities do you do in the **spring/summer?**

1.____________________ 4.____________________

2.____________________ 5.____________________

3.____________________

11. When you are **not** at school what sports or activities do you do in the **fall/winter?**

1. ____________________ 4.____________________

2.____________________ 5.____________________

3.____________________

12. **Do you play on any sports teams at school?**

Yes No (please circle one)

If **yes**, which teams do you play on?

____

____

ID #

13. Fill in the blanks with the words shown below.

Cardiorespiratory Fun Endurance Heart
Good 90
Pulse Strength Lungs

Sally goes for a 30 minute jog each day which increases her _____________ fitness.

Running every day is good for her ________ and ________.

Sally thinks that physical activity is _____ and is also _____ for her so she participates in at least ____ minutes a day.

When she goes to her sport team's practice she does exercises like push-ups and sit-ups that increase her ________ and _______.

Afterwards she checks her heart rate which is also called a ______.

14. Please circle all of the healthy foods. Put an X through the foods that are not good for you.

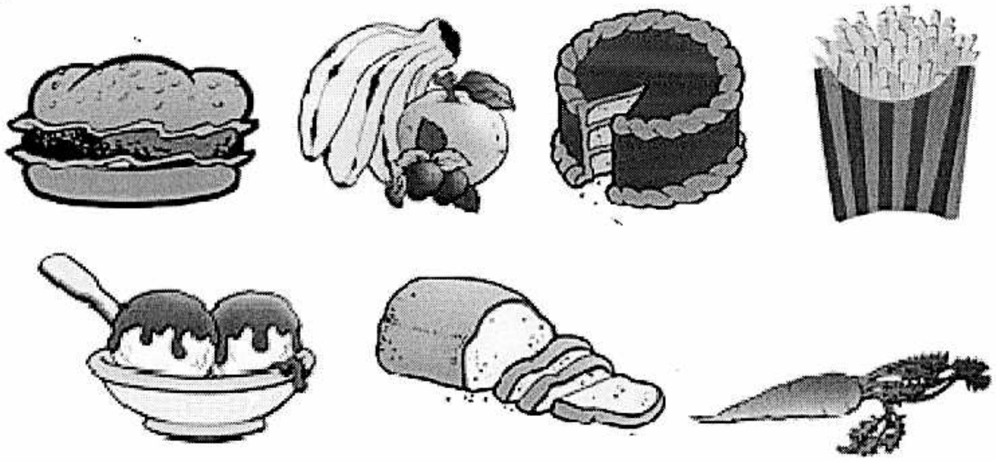

ID #

17. Do your brothers, sisters or parents participate in sports or physical activities? (play or coach).

Yes No (please circle one)

If **yes**, which sports do they participate in?

____

15. Please circle how you get to school <u>most of the time</u> in the fall/winter (during cold or snowy weather)

bus	walk	skate board	bike
car / truck	rollerblade	scooter	dog sled

How long does it take you to get to school in the fall/winter? __________

16. Please circle how you get to school <u>most of the time</u> in the spring/summer (during nice weather)

bus	walk	skate board	bike
car / truck	rollerblade	scooter	dog sled

How long does it take you to get to school in the spring/summer? __________

18. If you were allowed to pick what you do after school, which activity would you pick?

Play video/computer games

Read

Do homework

Play with my friends at the playground

Go to my sports team's practice

Walk my dog

Chat with friends online

Watch television

ID #

19. What do you think it means to be physically fit? What makes a person "fit" or "unfit"?

____

____

____

____

17. How much TV do you watch:

i. After school?
a. Less than 1 hour
b.1 to 2 hours
c. 3 to 4 hours
d. More than 5 hours
e. None

ii. *After dinner?*
a. Less than 1 hour
b.1 to 2 hours
c. 3 to 4 hours
d. More than 5 hours
e. None

iii. *On weekends?*
a. Less than 1 hour
b.1 to 2 hours
c. 3 to 4 hours
d. More than 5 hours
e. None

18. How much time do you spend on the computer:

i. After school?
a. Less than 1 hour
b.1 to 2 hours
c. 3 to 4 hours
d. More than 5 hours
e. None

ii. *After dinner?*
a. Less than 1 hour
b.1 to 2 hours
c. 3 to 4 hours
d. More than 5 hours
e. None

iii. *On weekends?*
a. Less than 1 hour
b.1 to 2 hours
c. 3 to 4 hours
d. More than 5 hours
e. None

☺ Thank you for your help! ☺
