# Supplementary material for: Physical Literacy Knowledge Questionnaire: feasibility, validity, and reliability for Canadian children aged 8 to 12 years
Source: BMC Public Health. 2018 Oct 2;18(Suppl 2):1035. doi: 10.1186/s12889-018-5890-y (PMC6167766; doi:10.1186/s12889-018-5890-y)
Supplement: Supplementary file 2 — Version 1 of the Cognitive domain questions (as of 2012-02-14). (DOCX 1747 kb) [file 12889_2018_5890_MOESM2_ESM.docx]

Physical Activity Knowledge

**(Canadian Assessment of Physical Literacy)**

**What grade are you in**:
1 2 3 4 5 6 7 8 **(please circle one)**

**Are you a**: boy girl **(please circle one)**

**What month is your birthday:** **(please circle one)**

Jan Feb Mar Apr May Jun Jul Aug Sept Oct Nov Dec

**How old are you**:
5 6 7 8 9 10 11 12 13 14 15
**(please circle one)**

**In this project, when we talk about physical activity, we mean when you are moving around, playing or exercising. Physical activity is any activity that makes your heart beat faster or makes you get out of breath some of the time.**

**Why are we asking you these questions? We want to know what kids like you think about physical activity, sports and exercise.**

**Please remember:**

- **There are no right or wrong answers. We only want to know what you think.**
- **If you do not know an answer, please write your best guess.**
- **There is no time limit, so please take all the time you need.**

1. **Being physically active means you are moving around and not sitting still. How important do you think it is that you are physically active every day?
   (circle one number)**Not important Somewhat important Very important
    1 2 3 4 5 6 7 8 9 10
2. **How important is it for you to be more active than you are now? (circle one number)**I’m already I need to be a I need to be
   active enough little more active a lot more active
    1 2 3 4 5 6 7 8 9 10
3. **Compared to other kids your age, how active are you? (circle one number)**A lot less active Same A lot more active
    1 2 3 4 5 6 7 8 9 10
4. **Compared to other kids your age, how good are you at sports or skills?
   (circle one number)**Others are better Same I’m a lot better
    1 2 3 4 5 6 7 8 9 10
5. **There are many different kinds of fitness. One type is called endurance fitness or aerobic fitness or cardiorespiratory fitness. Cardiorespiratory fitness means…
   (circle the right answer)**
6. How well the muscles can push, pull or stretch.
7. How well the heart can pump blood and the lungs can provide oxygen.
8. Having a healthy weight for our height.
9. Our ability to do sports that we like.
10. **Activities that make your heart beat faster and make you breathe faster, like walking fast or running, are called moderate or vigorous activity. How many minutes of moderate or vigorous physical activity are students supposed to do every day at school?**
11. 10 minutes
12. 20 minutes
13. 30 minutes
14. 60 minutes or 1 hour
15. **How many minutes of moderate or vigorous activity should you and other Canadian children do physical activity every day? Count the time you should be active at school and also the time you should be active at home or in your neighbourhood.**
16. 10 minutes
17. 20 minutes
18. 30 minutes
19. 60 minutes or 1 hour
20. **Sometimes children have to sit still to read, watch television or do homework. What is the most time that children should sit still each day? Do not count the time that you are asleep at night.**
21. 30 minutes
22. 60 minutes or 1 hour
23. 2 hours
24. 4 hours
25. **Draw a line to all the words you think describe what “Healthy” means.**

Not being sick

Exercising looking good

Eating well feeling good

Healthy is...

Being skinny Being flexible

Being attractive Being happy

Strong muscles Having endurance Being popular

1. **All of the athletes in the photos below are doing the same sport skill.**

**Which sport skill are they all doing?** ________________________________


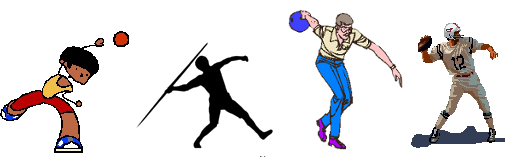


1. **This story about Sally is missing some words. Fill in the missing words below. Each word can only be used to fill one blank space in the story.**

**Cardiorespiratory Fun Endurance Heart Good 60**

**Pulse Strength Lungs**

Sally goes for a 30 minute jog each day which increases her ________________ fitness. Running every day is good for her _____________ and _____________.

Sally thinks that physical activity is _____________ and is also ____________ for her so she participates in at least _____________ minutes a day. At her sport team’s practice she does more running to improve her _________________. The team also does exercises like push-ups and sit-ups that increase her _________________. After exercising, she checks her heart rate which is also
called a _________________.

1. **In the summer, when you are outside for at least 30 minutes, how often do you wear sunscreen? (circle one answer)**a) Always
   b) Often
   c) Sometimes
   d) Not very often
   e) Never
2. **In the winter, when you are outside for at least 30 minutes, how often do you wear sunscreen? (circle one answer)**a) Always
   b) Often
   c) Sometimes
   d) Not very often
   e) Never
3. **Please circle all of the healthy foods. Put an X through the foods that
    are not good for you.**


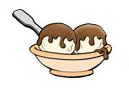

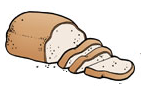

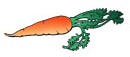

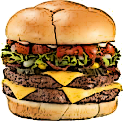

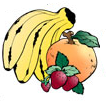

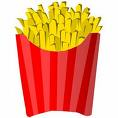

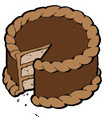


1. **How many hours do you usually spend sleeping each night?
   (circle one answer)**a) Less than 5 hours
   b) 5 to 8 hours
   c) 8 to 10 hours
   d) 10 to 12 hours
   e) More than 12 hours
2.
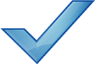
**Circle each activity that you do. If you always or almost always wear safety gear (like helmet or shin pads) when you do the activity, add a check mark inside the circle.**


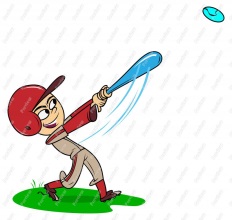

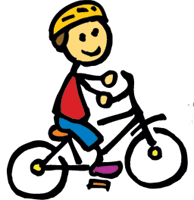

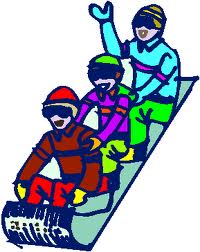

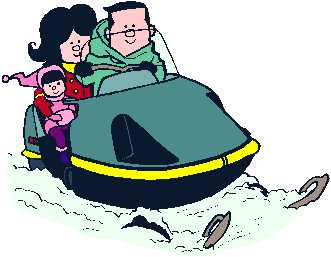

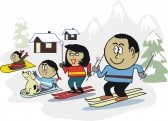

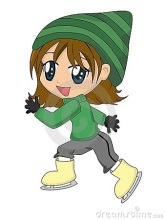

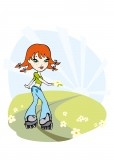

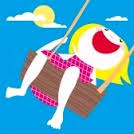

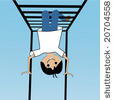

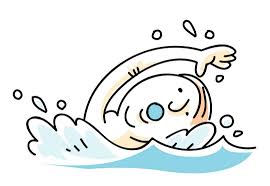

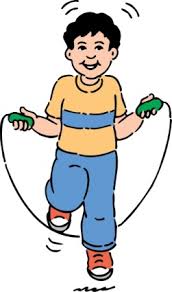


1. **If you wanted to get better at a sport skill like kicking and catching a ball, what would be the best thing to do?
   (circle one answer)**a) Read a book about kicking and catching a ball
   b) Wait until you get older
   c) Try kicking or catching a lot of times
   d) Take a lesson or have a coach teach you how to kick and catch
2. **If you wanted to get in better shape, what would be the best thing to do?
   (circle one answer)**a) Read a book about getting in shape
   b) Wait until you get older
   c) Try exercising or being active a lot more
   d) Take a lesson or have a coach teach you how to get in shape
3. **If you were allowed to pick what you do after school, which activity would you pick? (circle only one activity)**

Play video/computer games Go to my sports team’s practice

Read Walk my dog

Do homework Chat with friends online

Play with my friends at the playground Watch television
