# Supplementary material for: Physical Literacy Knowledge Questionnaire: feasibility, validity, and reliability for Canadian children aged 8 to 12 years
Source: BMC Public Health. 2018 Oct 2;18(Suppl 2):1035. doi: 10.1186/s12889-018-5890-y (PMC6167766; doi:10.1186/s12889-018-5890-y)
Supplement: Supplementary file 3 — Final version of the Physical Activity knowledge questions (as of 2013-06-28). (DOC 236 kb) [file 12889_2018_5890_MOESM3_ESM.doc]

# Physical Activity Questionnaire

**(Canadian Assessment of Physical Literacy)**

**What school grade are you in**:
1 2 3 4 5 6 7 8 **(please circle one)**

**Are you a**: boy girl **(please circle one)**

**What month is your birthday:** **(please circle one)**

Jan Feb Mar Apr May Jun Jul Aug Sept Oct Nov Dec

**How old are you**:
5 6 7 8 9 10 11 12 13 14 15
**(please circle one)**

**In this project, when we talk about physical activity, we mean when you are moving around, playing or exercising. Physical activity is any activity that makes your heart beat faster or makes you get out of breath some of the time.**

**Why are we asking you these questions? We want to know what kids like you think about physical activity, sports and exercise.**

**Please remember:**

- **There are no right or wrong answers. We only want to know what you think.**
- **If you do not know an answer, please write your best guess.**
- **There is no time limit, so please take all the time you need.**

1. **How many minutes each day should you and other children do physical activities that make your heart beat faster and make you breathe faster, like walking fast or running? Count the time you should be active at school and also the time you should be active at home or in your neighbourhood.**
2. 10 minutes
3. 20 minutes
4. 30 minutes
5. 60 minutes or 1 hour
6. **Compared to other kids your age, how active are you? (circle one number)**A lot less active Same A lot more active
    1 2 3 4 5 6 7 8 9 10
7. **Compared to other kids your age, how good are you at sports or skills?
    (circle one number)** Others are better Same I’m a lot better
    1 2 3 4 5 6 7 8 9 10
8. **Sometimes children watch television, play video games or play on the computer or on their smart phone. What is the most time that children should look at a screen each day? Do not count the time that you have to look at a screen to do your homework.**
9. 30 minutes
10. 60 minutes or 1 hour
11. 2 hours
12. 4 hours
13. **There are many different kinds of fitness. One type is called endurance fitness or aerobic fitness or cardiorespiratory fitness. Cardiorespiratory fitness means…
    (circle the right answer)**
14. How well the muscles can push, pull or stretch.
15. How well the heart can pump blood and the lungs can provide oxygen.
16. Having a healthy weight for our height.
17. Our ability to do sports that we like.
18. **Muscular strength or muscular endurance means…
    (circle the right answer)**
19. How well the muscles can push, pull or stretch.
20. How well the heart can pump blood and the lungs can provide oxygen.
21. Having a healthy weight for our height.
22. Our ability to do sports that we like.
23. **Draw a line to all the words you think describe what “Healthy” means.**

Being skinny looking good

Healthy is...

Eating well feeling good

Not being sick

1. **This story about Sally is missing some words. Fill in the missing words below. Each word can only be used to fill one blank space in the story.**

**Fun Endurance Good**

**Pulse Strength**

Sally tries to be active every day. Running every day is good for her heart and lungs.

Sally thinks that physical activity is _____________ and is also ____________ for her. At her sport team’s practice she does more running to improve her _________________. The team also does exercises like push-ups and sit-ups that increase her _________________. After exercising, she checks her heart rate which is also called a ______________.

9**. Circle each activity that you do. If you always or almost always wear safety gear (like helmet or shin pads) when you do the activity, add a check mark inside the circle.**


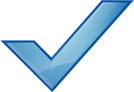


**10. If you wanted to get better at a sport skill like kicking and catching a ball, what would be the best thing to do?
(circle one answer)**


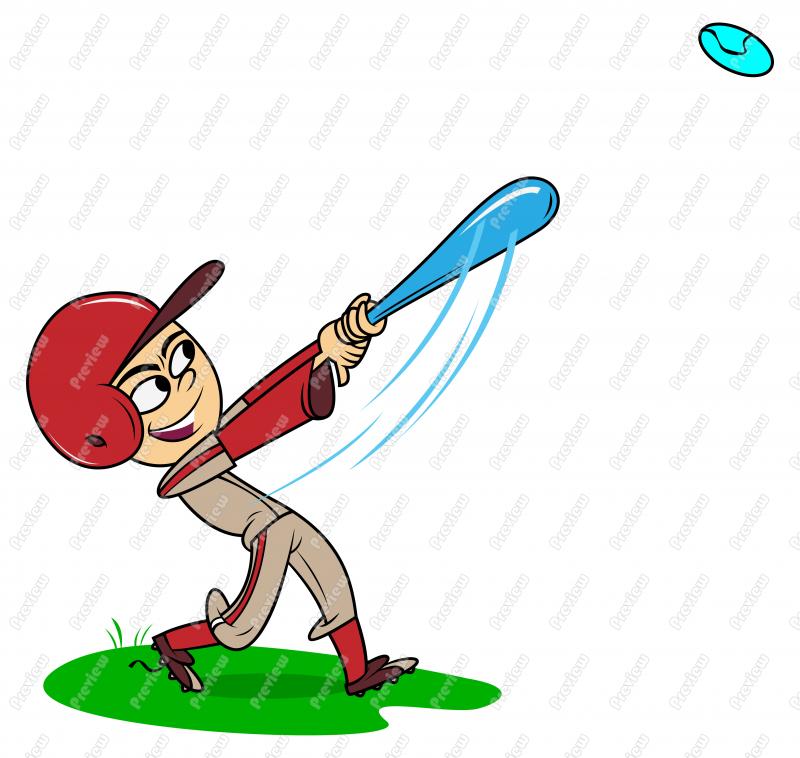

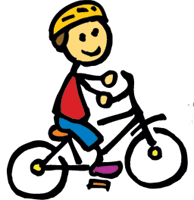

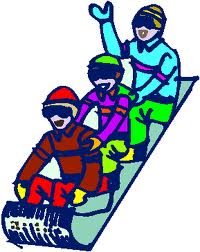

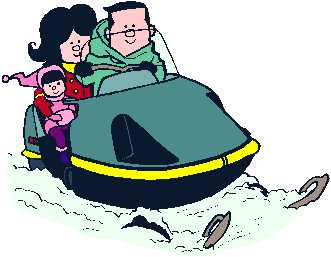

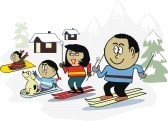

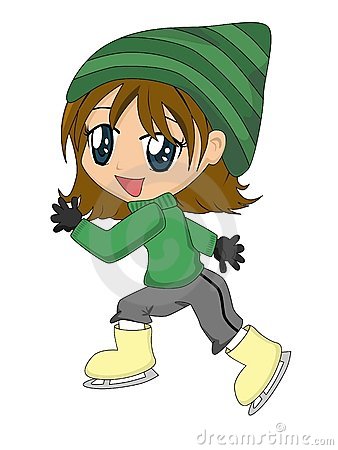

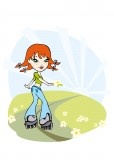

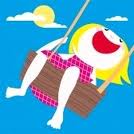

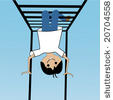

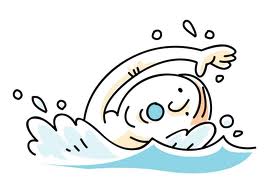

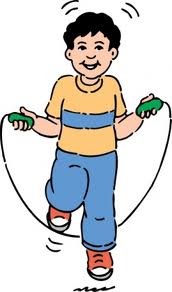


a) Read a book about kicking and catching a ball
b) Wait until you get older
c) Try exercising or being active a lot more
d) Watch a video, take a lesson or have a coach teach you how to kick and catch

**11. If you wanted to get in better shape, what would be the best thing to do?
(circle one answer)** a) Read a book about getting in shape
 b) Wait until you get older
 c) Try exercising or being active a lot more
 d) Watch a video, take a lesson or have a coach teach you how to get in shape

**12. If you were allowed to pick what you do after school, which activity would you pick? (circle only one activity)**

Play video/computer games Go to my sports team’s practice

Read Walk my dog

Do homework Chat with friends online

Play outside with my friends Watch television
